# Re_2_O_7_-catalyzed reaction of hemiacetals and aldehydes with *O*-, *S-,* and *C*-nucleophiles

**DOI:** 10.3762/bjoc.9.174

**Published:** 2013-07-30

**Authors:** Wantanee Sittiwong, Michael W Richardson, Charles E Schiaffo, Thomas J Fisher, Patrick H Dussault

**Affiliations:** 1 Department of Chemistry, University of Nebraska-Lincoln, Lincoln, NE 68588-0304, USA

**Keywords:** allylation, hemiacetal, *O*,*O-*acetal, *O*,*S-*thioacetal, peroxyacetal, Re_2_O_7_, *S*,*S*-acetal

## Abstract

Re(VII) oxides catalyze the acetalization, monoperoxyacetalization, monothioacetalization and allylation of hemiacetals. The reactions, which take place under mild conditions and at low catalyst loadings, can be conducted using hemiacetals, the corresponding *O*-silyl ethers, and, in some cases, the acetal dimers. Aldehydes react under similar conditions to furnish good yields of dithioacetals. Reactions of hemiacetals with nitrogen nucleophiles are unsuccessful. 1,2-Dioxolan-3-ols (peroxyhemiacetals) undergo Re(VII)-promoted etherification but not allylation. Hydroperoxyacetals (1-alkoxyhydroperoxides) undergo selective exchange of the alkoxide group in the presence of either Re_2_O_7_ or a Brønsted acid.

## Introduction

The synthetically important conversions of hemiacetals to acetals, thioacetals, or homoallyl ethers are typically achieved through activation of the substrate with a strong Brønsted or Lewis acid, or by conversion to an activated intermediate such as a haloacetal [1]. Perrhenates, best known as catalysts for large-scale alkene metathesis [2] and isomerization of allylic alcohols [3–10], have been shown to promote condensation of carbonyls with hydrogen peroxide or hydroperoxides [11–12], intramolecular displacements of reversibly formed hemiacetals and allylic alcohols [8,13], displacement of resonance-activated alcohols with electron-poor nitrogen nucleophiles [14], and a synthesis of homoallylated amines from condensation of carbonyl groups with an electron-poor amine in the presence of allyltrimethylsilane [15]. We now describe the Re_2_O_7_-promoted reactions of peroxyhemiacetals, hemiacetals, and alkoxyhydroperoxides with *O*-, *S-* and *C*-nucleophiles.

## Results

In the course of investigations into potential antischistosomal and antimalarial agents [16–17], we needed to prepare a number of 3-alkoxy-1,2-dioxolanes. Brønsted acid-promoted etherification of hemiacetals (1,2-dioxolan-3-ols) required harsh conditions and proceeded in good yields only for unhindered alcohols [18]. We were curious whether Re_2_O_7_, a catalyst known to activate alcohols via a reversibly formed Re(VII) ester [19–20], would enable displacement under milder conditions, potentially allowing access to a broader range of alkoxydioxolanes. As shown in Table 1, the use of Re_2_O_7_ or *p*-toluenesulfonic acid monohydrate (PTSA) as catalysts provided comparable yields in the etherification of dioxolanol **1a** or the corresponding *O*-trimethylsilyl ether **1b** with an unhindered alcohol, although the perrhenate-catalyzed reaction proceeded much more rapidly. In the case of a neopentyl alcohol nucleophile, the perrhenate-catalyzed process was clearly superior, proceeding more rapidly and furnishing a higher yield of acetal. The Re_2_O_7_-promoted reactions were subsequently found to proceed efficiently at only 1% catalyst loading. Neither catalyst allowed etherification with a tertiary alcohol.

**Table 1:** Acetalization of hydroxy- and silyloxydioxolanes.

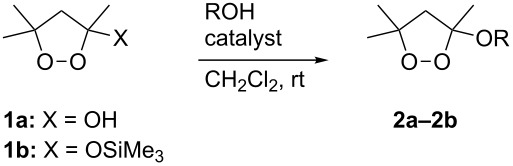					

R	substrate	product	catalyst (equiv)		

			PTSA (0.1)	Re_2_O_7_ (0.1)	Re_2_O_7_ (0.01)

			yield (reaction time)		

Ph(CH_2_)_2_	**1a**	**2a**	73% (3 h)	75% (1 h)	83% (1 h)
	**1b**	**2a**	—	—	81% (2 h)
1-AdCH_2_^a^	**1a**	**2b**	33% (12 h)	74% (1 h)	78% (2 h)
Ad^a^	**1a**	—	0%	0%	0%
	**1b**	—	—	—	0%

^a^Ad = adamantyl.

### Acetal formation

As illustrated in Table 2, we next investigated acetalization of tetrahydrofuranol **3**, tetrahydropyranol **4a** and the *O*-*t*-butyldimethylsilyl ether of the latter (**4b**). While good yields of acetals were obtained from the reaction with primary or secondary alcohols, or *t*-butyl hydroperoxide, acetalization with phenol proceeded in poor yield. Attempted acetalizations of 2,3,4,6-tetrabenzylglucose, the corresponding tetraacetate, or their 1-*O*-silyl derivatives, were unsuccessful (not shown).

**Table 2:** Transetherification of hemiacetals.

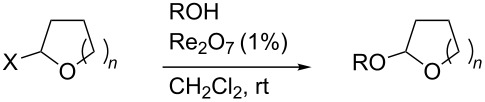						

substrate	*n*	X	R	equiv	product	yield^a^

**3**	1	OH	Ph(CH_2_)_2_	2	**5**	83%
**3**	1	OH	*t*-BuO (*t*-BuOOH)	2	**6**	68–77%
**4a**	2	OH	Ph(CH_2_)_2_	5	**7**	89%
**4b**	2	OTBS	Ph(CH_2_)_2_	2	**7**	82%
**4a**	2	OH	*t*-BuO	2	**8**	71–80%
**4a**	2	OH	2-octyl	2	**9**	70–90%
**4a**	2	OH	Ph	2	**10**	7%
**4b**	2	OTBS	Ph	1	**10**	44%^b^

^a^Duplicate values; range given in case of variance. ^b^Includes some inseparable impurities.

**Scheme 1:**
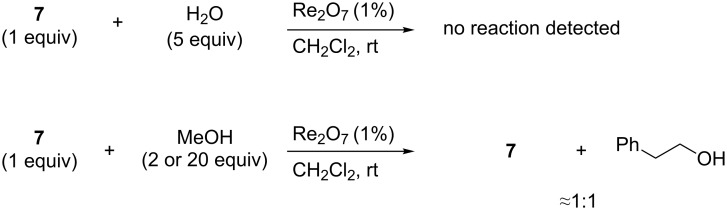
Transacetalization of acetal **7**.

Resubmission of purified acetal **7** to typical reaction conditions in the presence of a slight excess of water did not result in the reformation of **4a**. However, we did observe rapid transacetalization when **7** was resubmitted to reaction conditions in the presence of methanol (Scheme 1).

After TLC monitoring of reactions revealed the build-up of an unknown intermediate, we conducted control reactions in the absence of a nucleophile. These revealed a rapid Re_2_O_7_-promoted condensation of the hemiacetals to dimeric oxybisacetals (Table 3). As will be described later, these apparent byproducts proved to be competent substrates for acetal formation.

**Table 3:** Dimerization of hemiacetals.

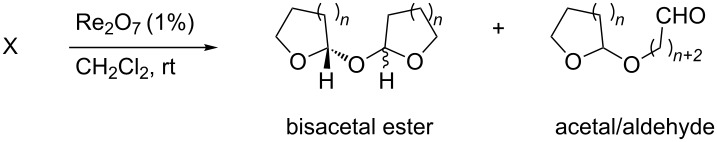						

entry	X	*n*	reaction time (h)	conditions^a^	bisacetal (%)	aldehyde (%)

1	**4a**	2	0.1	a	**11 (**57%)	**12** (5%)
2	**4a**	2	0.1	b	**11** (59%)	**12** (3%)
3	**4a**	2	0.5	a,d	**11** (12%)	trace
4	**4a**	2	2	a,e	NR	NR
5	**3**	1	0.1	a	**13** (37%)	**14** (28%)
6	**4a**	2	0.1	a	**11** (30%)	**12** (2%)
7	**4a**	2	0.5	c	**11** (62%)	**12** (4%)

^a^a:1% Re_2_O_7_; b: 2% Re_2_O_7_; c:10% PTSA; d: with 3 Å mol sieves (powdered); e: with 4 Å mol sieves (powdered).

We next attempted to maximize the yield of acetal based upon alcohol (Table 4). Good yields were obtained at a 1:1 ratio of alcohol to hemiacetal and yields did not vary significantly with the rate of addition of hemiacetal. This latter observation was initially surprising given the rapidity of hemiacetal dimerization (vide supra). However, we soon realized that the bisacetal ether **11** (Table 4, entry 4) was a remarkably effective substrate. In fact, the reaction of phenethyl alcohol (1.0 equiv) with only 0.5 equivalent of the oxybisacetal dimer proceeded rapidly to furnish high yields of **7** (not shown).

**Table 4:** Optimization of acetalization conditions.

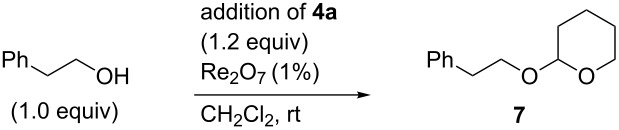					

entry	substrate	rate of **4a** addition (equiv/min)	reaction time (h)	additives	**7** (%)

1	**4a**	0.1	1	—	93
2	**4a**	0.04	1	—	94
3	**4a**	0.01	2	—	87
4	**11**	all at once	0.5	—	87
5	**4a**	all at once	1	—	89
6	**4a**	0.02	1	3 Å mol sieves (powder)	NR
7	**4a**	0.02	2	4 Å mol sieves (powder)	NR

### Monothioacetal formation

We next turned our attention to the synthesis of monothioacetals, a functionality important for carbonyl protection and as a precursor for oxycarbenium ions [21–22]. As illustrated in Table 5, tetrahydrofuranol and tetrahydropyranol both undergo condensation with a simple thiol in the presence of Re_2_O_7_ to provide the monothioacetal in good yield; only traces of the *S,S-*dithioacetal were observed. The same reaction, when catalyzed by a Brønsted acid, required a higher catalyst loading and provided a lower yield of product accompanied by a greater amount of dithioacetal. Re(VII)-promoted reaction with thioacetic acid proceeded in much lower yield.

**Table 5:** Synthesis of monothioacetals.

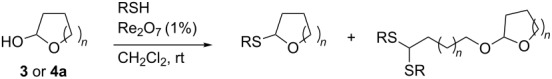					

substrate	catalyst	*n*	R	acetals	

				*O,S* (%)	*S,S* (%)

**3**	Re_2_O_7_	1	pentyl	**15** (71–80%)	(traces)
**4a**	Re_2_O_7_	2	pentyl	**16** (78–88%)	**17** (2%)
**4a**	Re_2_O_7_	2	acetyl	**18** (10%)	—
**4a**	PTSA	2	pentyl	**16** (62%)	**17** (8%)

**Scheme 2:**
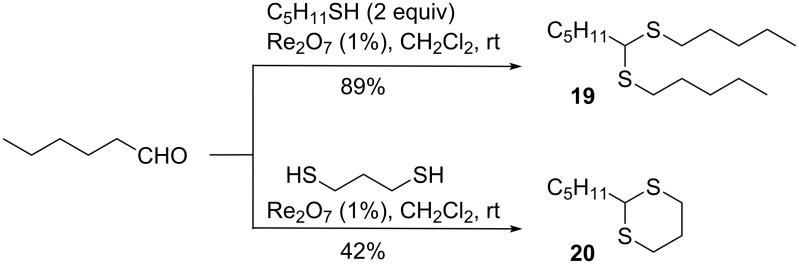
Thioacetalization of hexanal with Re_2_O_7_.

As illustrated in Scheme 2, Re_2_O_7_ also promotes the reaction of an aldehyde with a thiol or a dithiol to furnish a dithioacetal or a 1,3-dithiane. A ketone substrate did not react under these conditions (not shown).

The mono- and dithioacetalization reactions were visibly different from the *O-*acetalizations described above. Whereas addition of Re_2_O_7_ to a hemiacetal in the presence of an alcohol generates a transparent light yellow solution (Figure S1, Supporting Information File 1), addition of thiol to a mixture of Re_2_O_7_ and hemiacetal produces an opaque, black, solution that gradually becomes translucent but remains very dark (Figure S2, Supporting Information File 1).

### Attempted reaction with nitrogen nucleophiles

Perrhenate proved ineffective for catalyzing the formation of *N*,*O*-acetals (Table 6). Although these investigations were complicated by the limited solubility of some of the nucleophiles in dichloromethane, similar results were obtained in acetonitrile, where solubility was less of an issue.

**Table 6:** Attempted synthesis of *N*,*O*-acetals.

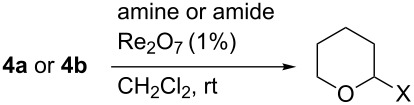			

substrate	Nu	X	product (yield)

**4a**	benzamide	BzNH	**21** (10%)
**4b**	benzamide	BzNH	—
**4a** or **4b**	acetamide	AcNH	—
**4a** or **4b**	benzylamine	BnNH	—
**4a** or **4b**	morpholine	morpholino	—

The poor results with *N-*nucleophiles led us to reinvestigate a proven reaction in the presence of amines. The previously successful condensation of **4a** with phenethyl alcohol (see Table 2) failed completely in the presence of added morpholine or pyridine (not shown).

### Allylation of hemiacetals

Re_2_O_7_ has been successfully applied to intramolecular Prins reactions of alkenes with reversibly generated hemiacetals [13], and we were curious about the potential for applications to intermolecular allylations. As illustrated in Table 7, tetrahydrofuranol **3**, tetrahydropyranol **4a**, bisacetal **11**, and oxadadamantyl hemiacetal **22** all underwent allylation in the presence of stoichiometric allyltrimethylsilane and 1% of Re_2_O_7._ The isolated yields of **23** and **24** may be artificially low due to product volatility. Peroxyhemiacetal (dioxolanol **1a**) failed to react under these conditions.

**Table 7:** Allylation of hemiacetals.

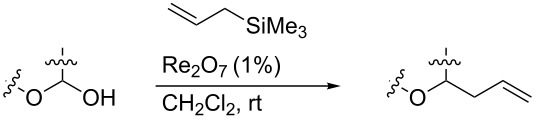		

substrate	product	yield

**1a**	NR	NR
**3**	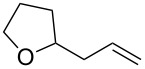 **23**^a^	33%^b^
**4a**	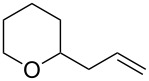 **24**^a^	41%^b^
**11**	**24**^a^	53%^c^
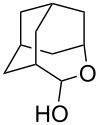 **22**	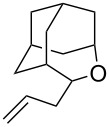 **25**	90%^c^

^a^Volatile product. ^b^Isolated yield. ^c^GC yield (toluene standard).

### Transetherification of hydroperoxyacetals

We were curious about the interactions of Re(VII) oxides with hydroperoxyacetals (1-alkoxyhydroperoxides), versatile intermediates available from ozonolysis of alkenes in alcoholic solvents [23–24]. As can be seen in Table 8, Re_2_O_7_, Me_3_SiOReO_3_ and PTSA all catalyze alkoxide metathesis to furnish a moderate yield of a new hydroperoxyacetal. The exchange reaction was observed in several solvents (e.g., CH_2_Cl_2_, 1,2-dichloroethane) but was most efficient in acetonitrile. The alkoxide exchange was accompanied by much slower exchange of the peroxide; for example, prolonged reaction (>24 h) of acetal **26** in MeOH (solvent) in the presence of catalytic Re(VII) furnished a 40% yield of the dimethyl acetal (not shown).

**Table 8:** Alkoxide exchange within hydroperoxyacetals.

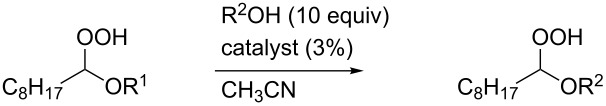						

substrate	R^1^	R^2^	catalyst	reaction time (h)	product	yield

**26**	Me	Et	Re_2_O_7_	2	**27**	41%
**26**	Me	Et	Me_3_SiOReO_3_	2	**27**	41%
**26**	Me	Et	PTSA	5	**27**	40%
**27**	Et	Me	Re_2_O_7_	2	**26**	43%
**26**	Me	iPr	Re_2_O_7_	2–48	mixtures	—
**28**	iPr	Me	Re_2_O_7_	0.5	**26**	42%

## Discussion

All the reactions described appear to involve the intermediacy of perrhenate esters. Previous investigators have hypothesized that the barrier for Re_2_O_7_-promoted C–O ionization of hemiacetals is relatively low [13]. The differences in reactivity between hemiacetals of cyclic ethers (displaced by alcohols and allyltrimethylsilane) and those of cyclic peroxides (reactive only towards alcohols) demonstrates that the extent of activation is dependent on the nature of the substrate, and that different levels of activation are required for trapping by heteroatom versus carbon nucleophiles. Previous work established that the perrhenate-catalyzed isomerization of alcohols can also employ silyl ethers as substrates [25], and our work demonstrates that the same is true for the hemiacetals investigated here. However, our work also demonstrates that oxybisacetals (for example, bis 2-tetrahydropyranyl ether) are highly effective substrates for the Re-catalyzed processes.

**Scheme 3:**
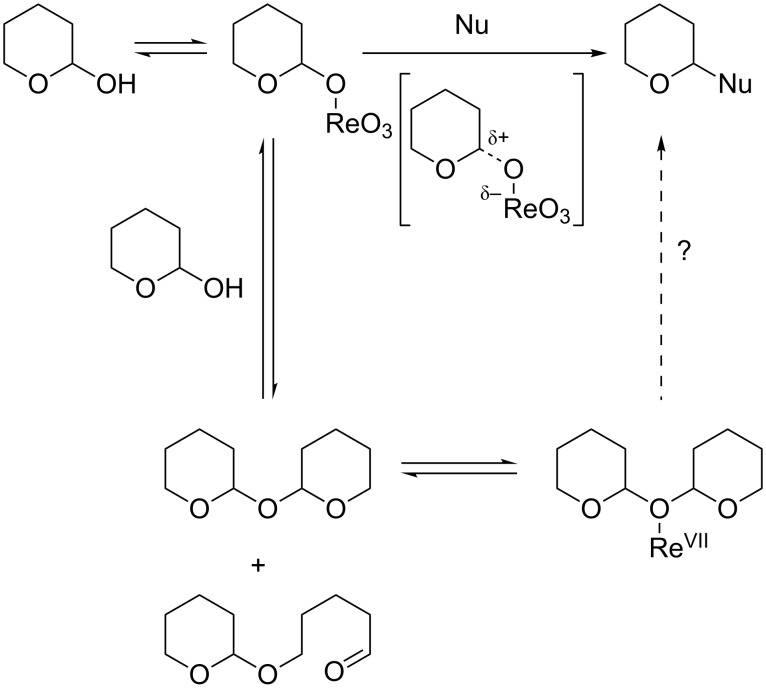
Proposed mechanistic pathway.

The results are in keeping with the formation of perrhenate esters which can undergo displacement by nucleophiles. Our results suggest that in the case of etherifications with alcohols, the intermediates can sometimes be regenerated from the product acetals. Moreover, our results clearly demonstrate that Re_2_O_7_ reversibly dimerizes hemiacetals in a reaction that is sufficiently rapid that it is possible that the oxybisacetal dimers may be the predominant precursors of the perrhenate intermediates and therefore the reaction products (Scheme 3).

The results with peroxyhemiacetals are consistent with the known reactivity of bisperoxyacetals [26]. The hydroperoxyacetals showed no sign of electrophilic activation of the hydroperoxide (which would presumably lead to heterolytic fragmentation) and activation of the peroxide C–O was clearly disfavored relative to activation of the alkoxide C–O bond. Rapid alkoxide metathesis was also observed in the presence of a strong Brønsted acid. Our observations suggest that the seeming lack of reactivity of ozonolysis-derived 1-methoxyhydroperoxides towards methanolic acid may mask a rapid degenerate exchange [27].

The failure of the perrhenate to catalyze formation of *N*,*O*-acetals was initially perplexing. However, reported perrhenate-promoted dehydrations of amides and oximes require relatively high temperatures [28–30]. Our results suggest that amines and amides actively suppress the ability of Re(VII)-oxides to activate alcohols. We note, however, a recent report by Ghorai describing the allylation of iminium ions generated from aldehydes and sulfonamides in the presence of Re_2_O_7_ [15].

## Conclusion

Perrhenates hold broad potential as catalysts for electrophilic activation of hemiacetals. The ability to catalyze formation of *O*,*O-*, *O*,*S-*, and *S*,*S-*acetals under very mild conditions offers a useful complement to traditional Brønsted acid catalysts.

## Supporting Information

Experimental details and detailed information, including references and spectral listings related to prepared molecules, are provided in Supporting Information File 1.

File 1 Experimental details.
