# Supplementary material for: Re2O7-catalyzed reaction of hemiacetals and aldehydes with O-, S-, and C-nucleophiles
Source: Beilstein J Org Chem. 2013 Jul 30;9:1526–32. doi: 10.3762/bjoc.9.174 (PMC3740507; doi:10.3762/bjoc.9.174)
Supplement: File 1 — Experimental details. [file Beilstein_J_Org_Chem-09-1526-s001.pdf]

Supporting Information

for

Re_2O_7-catalyzed reaction of hemiacetals and aldehydes with *O*-, *S*-, and *C*-nucleophiles

Wantanee Sittiwong, Michael W. Richardson, Charles E. Schiaffo, Thomas J. Fisher and Patrick H. Dussault*

Address: Department of Chemistry, University of Nebraska-Lincoln, Lincoln, NE 68588-0304, USA

Email: Patrick H. Dussault* - pdussault1@unl.edu

* Corresponding author

Experimental details

All reagents were used as received from commercial vendors, with the exception of CH_2Cl_2, which was distilled from CaH_2, and THF, which was distilled from sodium/benzophenone. Re_2O_7 (Strem, 99.9%) and $Me_3SiOReO_3$ (Gelest, 95%) were used as received. Reactions were conducted under an atmosphere of N_2 except where noted; flame-dried round bottom flasks (RBF) and 8 dram glass vials were used as reaction vessels. Thin layer chromatography (TLC) was performed on 0.25 mm hard-layer silica G plates; developed plates were visualized by staining: 1% ceric sulfate and 10% ammonium molybdate in 10% H_2SO_4 (general stain, after charring); 1% *N,N'*-dimethyl-*p*-phenylenediamine solution in 1:20:100 acetic acid/ water/ methanol (specific for peroxides) [1]; 1% aqueous $KMnO_4$ (for unsaturated compounds). "Standard drying and purification procedures" refers to drying of organic extracts over Na_2SO_4,

removal of solvent under vacuum, and purification by flash chromatography using the indicated eluent. $^{1}H/^{13}C$ NMR spectra were recorded at 300 or 400 MHz (^{1}H frequency) in $CDCl_3$; peaks are reported as: chemical shift (multiplicity, *J* couplings in Hz, number of protons).

General procedure A: To a vial containing a 0.2 M CH_2Cl_2 solution of the hemiacetal (1–1.2 equiv, **3** or **4a**) or the *O*-silyl ether (**4b**) was added nucleophile (1–10 equiv) followed by catalyst (Re_2O_7 or $TsOH{\cdot}H_2O$). After stirring for the indicated time (typically 1 h) the reaction was concentrated in vacuo and the residue purified via flash column chromatography (EA/hex or Et_2O/pentane). Reactions employing toluenesulfonic acid monohydrate as a catalyst were run in the same manner except that the crude reaction mixture was diluted with sat. aq. $NaHCO_3$ (≈4 mL/mmol substrate) and H_2O (≈4 mL/mmol substrate), and the mixture was extracted with ether (20 mL/mmol substrate, 2×). The combined organic layers were washed with brine (25 mL) and dried over Na_2SO_4. The residue was purified as described above.

General procedure B: A flame-dried round-bottom flask (RBF) was charged with catalyst followed by a 0.2 M solution of the acetal/hemiacetal (1 equiv) and nucleophile (2–10 equiv) in dry CH_2Cl_2. The reaction was stirred for the indicated time (typically 1 h) and then concentrated in vacuo. The residue was purified by flash column chromatography (EA/hex or Et_2O/pentane) as described above.

General procedure for Table 4: A flame-dried RBF was charged with catalyst (1 mol %) and 2-phenylethanol (1 equiv), after which a 0.2 M solution of hemiacetal **4a** (1.2 equiv) in CH_2Cl_2 was added at the indicated rate using a syringe pump. The reaction was stirred for the indicated time and then concentrated in vacuo. The residue was purified by flash column chromatography using 5% EA/hex.

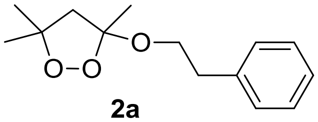

2a

5-(2-Phenylethoxy)-3,3,5-trimethyl-1,2-dioxolane (2a) was prepared from 3,3,5-trimethyl-1,2-dioxolan-3-ol (**1a**, 190.9 mg, 1.44 mmol), 1-phenylethanol (22 mL, 183 mmol) and toluenesulfonic acid monohydrate ($TsOH{\cdot}H_2O$, 26.3 mg, 0.14 mmol) using general procedure A (Brønsted acid-catalysis), which is a variant of a procedure previously described for this

transformation [2]. The reaction was stirred for 5 h and subjected to standard workup and purification (5% EA/hex) to afford acetal **2a** as a yellow oil (234 mg, 73%). R_f (5% EA/hex) 0.46. The spectral properties matched those previously reported [3].

Re_2O_7-catalyzed (1% loading): **2a** was prepared from **1a** (175.6 mg, 1.3 mmol), 1-phenylethanol (0.8 mL, 6.7 mmol), and Re_2O_7 (6.4 mg, 0.013 mmol) in CH_2Cl_2 (13 mL) using general procedure A. The reaction was allowed to run for 1 h and then subjected to standard workup and purification (10% EA/hex) to afford **2a** as a yellow oil (262.1 mg, 83%); the product was identical to the material described above.

2a could also be prepared from 3,5,5-trimethyl-1,2-dioxolan-3-ol-*O*-trimethylsilyl ether (**1b**), which was prepared by a reported procedure.[3]

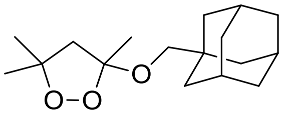

2b

3-(Adamant-1-ylmethoxy)-3,5,5-trimethyl-1,2-dioxolane (**2b**) was prepared from 1-adamantane methanol and **1a** or **1b** by similar procedures as described for **2a** to afford **2b** (164 mg, 0.79 mmol, 33% yield, 10% PTSA), (0.33 g, 1.2 mmol, 78% yield, 1% Re_2O_7); (0.32 g, 1.1 mmol, 74% yield, 10% Re_2O_7). Spectral properties matched those in a previous report [3].

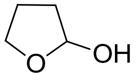

3

Tetrahydrofuran-2-ol (3) was prepared using a literature procedure; spectral properties matched those previously reported [4].

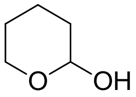

4a

Tetrahydro-2*H*-pyran-2-ol (4a) was prepared using a literature procedure; spectral properties matched those previously reported [5].

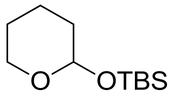

4b

***tert*-Butyldimethyl((tetrahydro-2*H*-pyran-2-yl)oxy)silane (4b):** Into a solution of imidazole (408 mg, 6.00 mmol) in THF (45 mL) in a flame-dried RBF was added **4a** (510 mg, 5.00 mmol), followed by a solution of TBSCl (900 mg, 6.00 mmol) in THF (5 mL). The reaction was stirred overnight and then quenched with H_2O (30 mL). The diethyl ether extract (3 × 10 mL) was subjected to standard drying and purification (10% EA/hex) to afford **4b** (287 mg, 13.3%). R_f (10% EA/hex): 0.48. ^{1}H NMR (400 MHz): 4.91 (m, 1H), 3.99 (m, 1H), 3.51 (m, 1H), 1.89-1.68 (6H), 0.91 (s, 9H), 0.12 (s, 3H), 0.90 (s, 3H). ^{13}C NMR (100 MHz): 90.1, 62.8, 33.4, 25.7, 25.5, 19.7, 18.1, −4.4, −5.4. HRMS (ESI): calc for $C_{11}H_{24}O_2Na$ 239.1443, found 239.1441 [6].

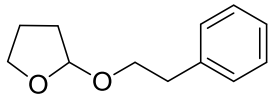

5

2-Phenethoxytetrahydrofuran (5) was prepared according to general procedure B using **3** (44 mg, 0.50 mmol), 2-phenylethanol (244 mg, 2.00 mmol), and Re_2O_7 (2.5 mg, 0.005 mmol) in CH_2Cl_2 (5 mL). The reaction was stirred for 1 h and the product was isolated and purified using standard procedures (5% EA/hex) to afford **48** (73.1 mg, 83.1%). R_f (25% EA/hex) 0.7. Spectral properties matched those in a previous report [7].

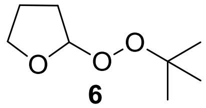

6

2-(*tert*-Butylperoxy)tetrahydrofuran (6): Into a vial containing a solution of Re_2O_7 (2.5 mg, 0.0050 mmol) in CH_2Cl_2 (3 mL) was added a decane solution of *t*-butylhydroperoxide (0.18 mL, 1.0 mmol, nominally 5.5 M), followed immediately by **4a** (44 mg, 0.50 mmol) neat. The reaction was stirred for 1 hour, then washed with 5 mL H_2O and extracted with CH_2Cl_2 (3 × 5 mL). Standard procedures for drying and purification (2.5% EA/hex) afforded **51** (54.4 mg, 68.0%). R_f (5% EA/hex) 0.29. Spectral properties matched those in a previous report [8].

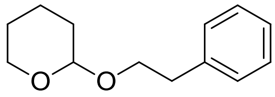

7

2-(2-Phenylethoxy)-tetrahydro-2*H*-pyran (7) could be prepared using general procedure A, B or C. Following a standard workup, the product was purified by column chromatography with 10% EA/hex. R_f (10% EA/hex) 0.41. The NMR spectra matched those previously reported [9].

Figure S1: Reaction solution for Re(VII)-catalyzed etherification of **4a** with phenylethanol.

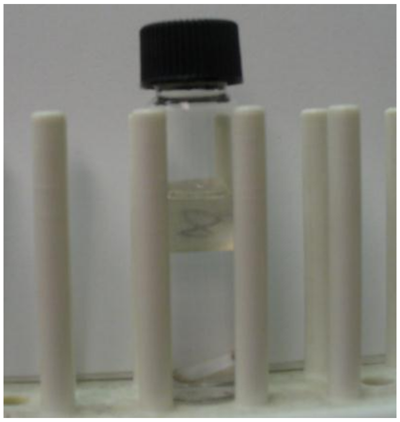

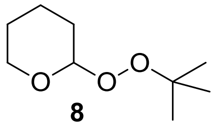

8

2-(*tert*-Butylperoxy)tetrahydro-2*H*-pyran (8) was prepared as described for **6** using **4a** (44 mg, 0.50 mmol), 5.5M (in deacane) *t*-butylhydroperoxide (0.18 mL, 1.0 mmol), and Re_2O_7 (2.5 mg, 0.0050 mmol) in CH_2Cl_2 (3 mL). The reaction was stirred for 1 h and was subjected to standard drying and purification procedures with 30% EA/hex to afford **51** (54.4 mg, 68%). R_f (30% EA/hex) 0.62. ^{1}H NMR (400 MHz) 5.07 (m, 1H), 4.05 (m, 1H), 3.63 (m, 1H), 1.83–1.71 (2H), 1.66–1.53 (4H), 1.30 (s, 9H). ^{13}C NMR (100 MHz): 101.2, 80.5, 62.7, 27.7, 26.4, 25.1, 20.0. HRMS (ESI): calc for $C_9H_{18}O_3Na$ 197.1154, found 197.1161 [10].

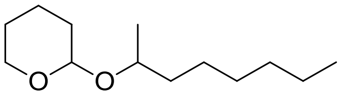

9

2-(Octan-2-yloxy)tetrahydro-2*H*-pyran (9) was prepared according to general procedures A or B using a solution of **4a** (102 mg, 1.00 mmol), 2-octanol (0.63 mL, 4.0 mmol), and Re_2O_7 (5.0 mg, 0.010 mmol) in CH_2Cl_2 (5 mL). The reaction was stirred for 1 h and the product was purified by flash column chromatography with 20% EA/hex to afford **9** (150 mg, 70.0%). R_f (20% EA/hex) 0.52. Spectral properties matched those previously reported [11].

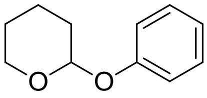

10

2-Phenoxytetrahydro-2*H*-pyran (**10**): (CAS 4203-50-3) was prepared according to general procedure A using a solution of **4a** (51 mg, 0.5 mmol), phenol (92 mg, 1.0 mmol) and Re_2O_7 (2.5 mg, 0.05 mmol) in CH_2Cl_2 (3 mL). The reaction was stirred for 1 h and the product was purified by flash column chromatography with 10% EA/hex to afford **10** (6 mg, 7%); or using a solution of **4b** (51 mg, 0.24 mmol), phenol (44 mg, 0.5 mmol) and Re_2O_7 (2.5 mg, 0.05 mmol) in CH_2Cl_2 (1.5 mL), with standard work up and purification to afford **10** with inseparable impurity (19 mg, 44%). R_f (10% EA/hex) 0.55. ^{1}H NMR (400 MHz): 7.35–7.25 (m, 2H), 7.07 (m, 2H), 7.00 (m, 1H), 5.44 (t, J = 3.3, 1H), 3.95 (ddd, J = 11, 9.4 and 1.2, 1H), 3.63 (ddt, J = 11, 4.3 and 1.5, 1H), 2.10–1.97 (m, 1H), 1.95–1.85 (m, 1H), 1.80–1.58 (m, 3H). ^{13}C NMR (100 MHz): 157.1, 129.4, 121.6, 116.5, 96.3, 62.1, 30.4, 25.2, 18.8.

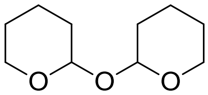

11

2,2'-Oxybis(tetrahydro-2*H*-pyran) (**11**): To a solution of **4a** (51 mg, 0.5 mmol) in CH_2Cl_2 (1 mL) was added Re_2O_7 (1 mg, ≈0.01 mmol). The reaction was stirred for 5 min and then concentrated in vacuo. The residue was purified by gradient flash column chromatography (2.5–15% Et_2O/pentane) to afford two diastomers of bisacetal **11** (major, 25.5 mg, 54%; minor 1.2 mg, 3%) accompanied by aldehyde **12** (2.5 mg, 5%).

11-*Major:* R_f (10% EA/hex) 0.45. ^{1}H NMR (400 MHz): 4.95 (dd, *J* = 4.9 and 2.5, 2H), 3.89 (ddd, *J* = 11, 7.5 and 3.3, 2H), 3.52 (m, 2H), 1.92–1.80 (m, 2H), 1.80–1.70 (m, 2H), 1.65–1.48 (m, 8H). ^{13}C (100 MHz): 94.6, 62.9, 30.6, 25.4, 19.7.

11-*Minor:* R_f (10% EA/hex) 0.33. ^{1}H NMR (400 MHz): 4.84 (dd, *J* = 4.5 and 2.9, 2H), 4.05 (m, 2H), 3.58 (m, 2H), 1.92–1.50 (m, 12H). ^{13}C NMR (100 MHz): 98.5, 63.4, 31.0, 25.4, 19.8.

Aldehyde **12**: R_f (10% EA/hex) 0.20. Spectral properties matched those previously reported [12].

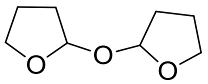

13

2,2'-Oxybis(tetrahydrofuran) (**13**) was prepared as described for **11.** The products were purified by gradient flash column chromatography (5–20% Et_2O/pentane) to afford major isomer **13** (45.7 mg, 30% yield) and 54.5 mg of a 4:1 mixture of aldehyde **14** and minor isomer **13** (7% and 28% yield based on ^{1}H NMR).

13-*Major:* R_f (10% EA/hex) 0.5; ^{1}H NMR (400 MHz): 5.44 (dd, *J* = 4.4 and 1.2, 2H), 3.95–3.82 (m, 4H), 2.05–1.78 (m, 8H). ^{13}C NMR (100 MHz): 100.0, 67.0, 32.3, 23.4. 0.50, IR (ZnSe): 2953, 2875, 1108, 986, 921 cm^{-1}.

Aldehyde **14** and **13-***Minor*: R_f (10% EA/hex) 0.11; Spectral properties of aldehyde **14** matched those previously reported [13].

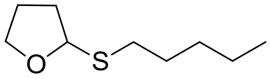

15

2-(Pentylthio)tetrahydrofuran (15) was prepared according to general procedure A or B. Standard workup and purification (3% and 5% EA/hex) afforded **15** (61.6 mg, 0.35 mmol, 71% yield) [14]. R_f (10% EA/hex) 0.61. ^{1}H NMR (400 MHz): 4.62 (m, 1H), 3.76 (m, 2H), 2.54 (m, 2H), 1.83–1.63 (6H), 1.39–1.26 (4H), 0.91 (t, *J* = 6.92, 3H). ^{13}C NMR (100 MHz): 84.4, 66.7, 32.6, 31.2, 29.7, 24.8, 22.3, 14.0. HRMS (ESI): calc for $C_9H_{18}OSNa$ 197.0976, found 197.0984.

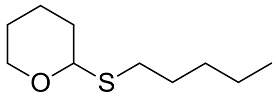

16

2-(Pentylthio)tetrahydro-2*H*-pyran (16) was prepared according to general procedure A or B. Standard workup and purification (2.5% EA/hex) afforded thioacetal **16** (73 mg, 0.39 mmol, 78% yield, 1% Re_2O_7); (43.4 mg, 0.23 mmol, 62% yield, 10% PTSA); and a trace amount of aceta/dithioacetal **17** (1.7 mg, 0.0045 mmol, 2% yield, 1% Re_2O_7) and (7.4 mg, 0.02 mmol, 8% yield, 10% PTSA). R_f (10% EA/hex) 0.66. ^{1}H NMR (400 MHz) 4.86 (m, 1H), 4.11 (m, 1H), 3.52 (m, 1H), 2.63 (m, 2H), 1.94 (m, 1H), 1.84 (m, 1H), 1.73–1.53 (6H), 1.43-1.29 (4H), 0.91 (t, J = 6.92, 3H). ^{13}C NMR (100 MHz): 82.1, 64.5, 31.4, 31.1, 30.3, 29.6, 25.6, 22.2, 21.8, 13.9. HRMS (ESI): calc for $C_{10}H_{20}OSNa$ 211.1133, found 211.1130 [14,15].

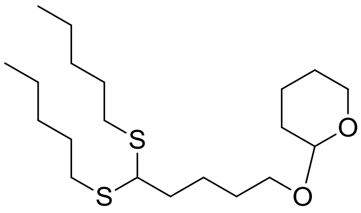

17

2-((5,5-bis(pentylthio)pentyl)oxy)tetrahydro-2*H*-pyran (**17**): R_f (10% EA/hex) 0.58. IR (ZnSe): 2924, 2858, 1120, 1034 cm^{-1}. ^{1}H NMR (400 MHz) 4.60 (m, 1H), 3.89 (m, 1H), 3.76 (t, *J* = 6.8, 1H), 3.75 (m, 1H), 3.52 (m, 1H), 3.41 (m, 1H), 2.67 (dt, *J* = 12.4, 7.4, 2H), 2.57 (dt, *J* = 12.4, 7.4, 2H), 1.90–1.81 (m, 2H), 1.78–1.69 (m, 1H), 1.67–1.50 (m, 12H), 1.43–1.29 (m, 8H), 0.91 (t, *J* = 7.2, 6H). ^{13}C NMR (100 MHz): 98.9, 67.3, 62.3, 51.6, 36.0, 31.2, 30.8, 30.1, 29.3, 29.1, 25.5, 24.3, 22.3, 19.7, 14.0. HRMS (ESI): calc for $C_{20}H_{40}O_2S_2Na$ 399.2367, found 399.2355.

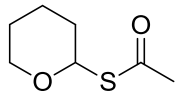

18

Tetrahydro-2*H*-pyran-2-thiol, acetate ester (**18**) was prepared according to general procedure A or B using **4a** (65 mg, 0.64 mmol), thioacetic acid (76 mg, 1.0 mmol), and Re_2O_7 (2.5 mg, 0.0052 mmol) in CH_2Cl_2 (3 mL). After reaction for 1h, standard workup and purification (10% EA/hex) furnished **18** (10 mg, 10% yield). R_f (20% EA/hex) 0.51. Spectral properties matched those from literature reports [16].

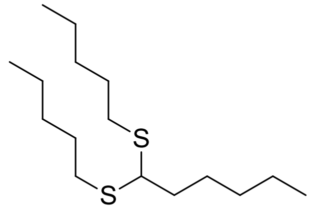

19

1,1-Bisthiopentylhexane (**19**)**:** To a solution of hexanal (50 mg, 0.5 mmol) and pentanethiol (0.13 mL, 1.0 mmol) in CH_2Cl_2 (3 mL) was added Re_2O_7 (2.5 mg, 0.05 mmol). The reaction was stirred for 30 min and then concentrated in vacuo. The residue was purified by flash column chromatography (hexane) to afford **19** (0.1286 g, 89%). R_f (hex) 0.22. IR (ZnSe): 2955, 2924, 2857, 1465, 732 cm^{-1}. ^{1}H NMR (400 MHz): 3.76 (t, *J* = 9.2, 1H), 2.68 (dt, *J* = 16.4 and 10, 2H), 2.57 (dt, *J* = 16.4 and 10, 2H), 1.80 (m, 2H), 1.70–1.22 (m, 19H), 0.92 (m, 9H). ^{13}C NMR (100 MHz): 52.1, 36.1, 31.4, 31.2, 30.1, 29.1, 27.2, 22.5, 22.3, 14.01, 13.96. HRMS (ESI): calc for $C_{16}H_{34}S_2Na$ 313.2000, found 313.1998.

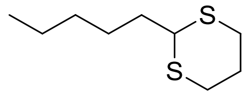

20

2-Pentyl-1,3-dithiane (**20**) was prepared as described for **19** using hexanal (50 mg, 0.5 mmol) and 1,3-propanedithiol (0.06 mL, 0.6 mmol) in CH_2Cl_2 (3 mL) and Re_2O_7 (2.5 mg, 0.05 mmol) to afford **20** (40 mg, 42% yield) R_f (hex) 0.23. Spectral properties matched those previously reported [17].

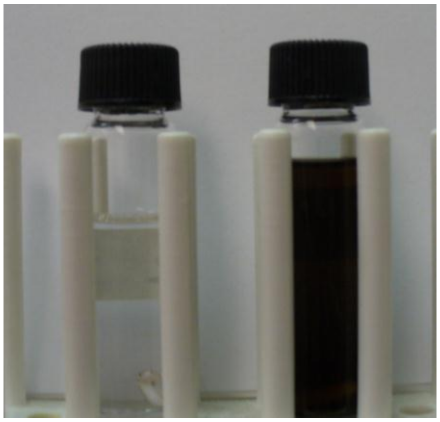

Figure S2: Reaction solutions for Re(VII)-catalyzed etherification of **4a** with phenylethanol (left) vs pentanethiol (right).

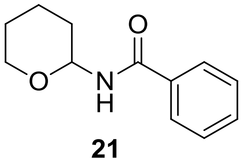

21

***N*-(Tetrahydro-2*H*-pyran-2-yl)benzamide** (**21**) was prepared according to general procedure A using **4a** (51 mg, 0.5 mmol), benzamide (0.30 mg, 2.5 mmol), and Re_2O_7 (2.5 mg, 0.005 mmol) in CH_2Cl_2 (5 mL). The reaction was stirred for 2 h. The product was purified by column chromatography with 40% EA/hex and after evaporation of the solvent the resulting solid was washed with 20% EA/hex (1 mL × 2) to afford **21** (10 mg, 10% yield). R_f (20%EA/hex) 0.51. Spectral properties matched those previously reported [18].

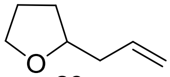

23

2-Allyltetrahydrofuran (**23**) was prepared according to general procedure A or B using **4a** (44 mg, 0.50 mmol), allyltrimethylsilane (0.79 mL, 2.50 mmol), and Re_2O_7 (2.5 mg, 0.0050 mmol) in CH_2Cl_2 (3mL). The reaction was stirred for 4 h and the product was purified with 10% Et_2O/pentane to afford **23** (19 mg, 33% yield). R_f (20%EA/hex) 0.52. Spectra matched literature reports [4].

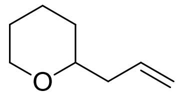

24

2-Allyltetrahydro-2*H*-pyran (**24**) was prepared general procedure A or B using **4a** (102 mg, 1.00 mmol), allyltrimethyl silane (1.58 mL, 5.00 mmol), and Re_2O_7 (5.0 mg, 0.0010 mmol) in CH_2Cl_2 (5mL). The reaction was stirred for 4 h and the product was purified by column chromatography with 10% Et_2O/pentane to afford **24** (26 mg, 41% yield). R_f (20%EA/hex) 0.54. Spectra matched literature reports [19].

4-Oxatricyclo[4.3.1.1^{3,8}]undecan-5-ol (22): To a flame-dried RBF containing a solution of 2-adamantanone (3.0 g, 20 mmol) in CH_2Cl_2 (100 mL) was added sequentially *m*-chloroperoxybenzoic acid (7.15 g, 25 mmol), 20 mL of water and 20 mL of sat. $NaHCO_3$. After stirring for 24 h, the reaction was quenched with Na_2SO_3 (1 g) and then washed sequentially with sat. aq. $NaHCO_3$ (20 mL) and brine (20 mL). The combined organic layers were dried with $MgSO_4$, and the residue obtained upon concentration was purified by column chromatography with 60% EA/hex to afford 4-oxatricyclo[4.3.1.1^{3,8}]-undecan-5-one (2.8522 g, 86%) as a white solid. R_f (60% EA/hex):0.48. Spectral properties matched those reported [20].

To a flame dried flask containing a −78 °C solution of 4-oxatricyclo[4.3.1.1^{3,8}]undecan-5-one (2.0 g, 11 mmol) in CH_2Cl_2 (60 mL) was dropwise added a solution of DIBAL-H in toluene (8.3 mL, 12.5 mmol, nominally 1.5 M). The reaction was stirred at −78 °C for 1 h and then quenched with MeOH (0.5 mL). The reaction was allowed to warm to rt, and then stirred for 45 min. A solution of sat. aq. Rochelle's salt (30 mL) was added and the resulting suspension was extracted with CH_2Cl_2 (3 × 40 mL). The combined organic layers were washed with H_2O (40 mL) and dried with $MgSO_4$. The residue obtained upon concentration was subjected to chromatography with 40 % EA/hex to afford hemiacetal **22** (1.9104 g, quant) as a white solid: mp 254–260 °C (uncorrected; literature 243–252) [17]; R_f (40 % EA/hex) 0.43. The NMR spectra matched those previously reported [21].

5-Allyl-4-oxatricyclo[4.3.1.1^{3,8}]undecane (25) was prepared according to general procedure A using oxadamantyl hemiacetal **22** (168.4 mg, 1 mmol), allyltrimethylsilane (0.8 mL, 5 mmol) and Re_2O_7 (4.8 mg, 0.01 mmol) in CH_2Cl_2 (10 mL). The reaction was stirred for 1 h and then treated with sat. aq. $NaHCO_3$ (5 mL) and H_2O (5 mL). The suspension was extracted with CH_2Cl_2 (3 × 20 mL) and the combined organic layers were dried with Na_2SO_4. The residue obtained upon concentration was subjected to chromatography (initially 10% EA/hex; repeat with 5% EA/hex) to afford **25** (166.5 mg, 90%). R_f (10% EA/hex) 0.6; ^{1}H NMR (400 MHz): 5.83 (m, 1H), 5.10–5.00 (2H), 4.18 (m, 1H), 3.81 (t, J = 6.8, 1H), 2.37 (m, 1H), 2.18 (m, 1H), 2.00–1.64 (10H), 1.61–1.49 (3H). ^{13}C NMR (100 MHz): 136.6, 116.5, 81.9, 71.8, 41.5, 40.9, 40.0, 26.1, 36.0, 34.6, 30.0, 26.9, 26.4. HRMS (EI): calc for $C_{13}H_{20}O$ 192.1514, found: 192.1519.

1-Methoxynonyl hydroperoxide (26), **1-ethoxynonyl hydroperoxide (27)**, and **1-isopropoxynonyl hydroperoxide (28)** were prepared as previously described [22].

Procedure for Re_2O_7 and Brønsted acid catalyzed exchange of hydroperoxyacetals:

A flame-dried 8 mL vial was charged with catalyst, followed by a 0.2 M solution of the starting material (1 equiv) and alcohol nucleophile (10 equiv) in dry acetonitrile. The reaction was stirred for the indicated time (typically 2–5 h) and then filtered through a short silica plug (ethyl acetate/hexane rinse). The resulting solution was concentrated in vacuo and the residue purified by flash chromatography (ethyl acetate/hexane). The spectra of products **26**, **27** and **28** matched literature reports [22].

References

(1) Smith, L, Hill, F. L. *J. Chrom.* **1972**, *66*, 101.
(2) Dussault, P. H.; Liu, X. *Org. Lett.* **1999**, *1*, 1391.
(3) Schiaffo, C. E.; Rottman, M.; Wittlin, S.; Dussault, P. H. *ACS Med. Chem. Lett.* **2011**, *2*, 316-319
(4) Saito, T.; Nishimoto, Y.; Yasuda, M.; Baba, A., *J. Org. Chem.*, **2006**, *71*, 8516.
(5) Kimura, M.; Shimizu, M.; Tanaka, S.; Tamaru, Y. *Tetrahedron* **2005**, *61*, 3709.

(6) Reported without details: McDonald, C. E.; Beebe, T. R.; Beard M.; McMillen, D.; Selski, D. *Tetrahedron Lett.* **1989**, *30*, 4791.
(7) Baati, R.; Valleix, A.; Mioskowski, C.; Barma, D. K.; Falck, J. R. *Org. Lett.* **2000**, *2*, 485.
(8) Nikalje, M. D.; Sudalai, A. *Tetrahedron* **1999**, *55*, 5903.
(9) Hajipour, A. R.; Nasresfahani, Z. *Synth. Commun.* **2012**, *42*, 1995; Williams, D. B. G.; Simelane, S. B.; Lawton, M.; Kinfe, H. H. *Tetrahedron* **2010**, *66*, 4573.
(10) Rigaudy, J.; Izoret, G. *Compt. Rend*. **1953**, *236*, 2086.
(11) Matsumoto, Y.; Mita, K.; Hashimoto, K.; Iio, H; Tokoroyama, T. *Tetrahedron* **1996**, *52*, 9387.
(12) Clasby, M. C.; Craig, D.; Slawin, A. M. Z.;White, A. J. P.; Williams, D. J. *Tetrahedron*, **1995**, *51*, 1509.
(13) Guy, F.; Balme, G.; Barieux, J. J.; Gore, J. *Tetrahedron*, **1988**, *44*, 5821.
(14) Zorin,V. V.; Batyrvaev, N. A.; Zlotskii, S. S.; Rakhmankulov, D. L. *Zh. Org. Khim.* (Russ. J Org. Chem.) **1984**, 20, 387.
(15) Cerny, M.; Trka, A. *Coll. of Czech. Chem. Comm.* **1983**, *48*, 1749; Eliel, E. L.; Nowak, B. E.; Daignault, R. A. *J. Org. Chem.* **1965**, *30*, 2448.
(16) Kansikas, J.; Leskela, M.; Sipila, K.; Hase, T. *Acta Chem. Scand.* **1995**, *49*, 809; Martin, M.; Bassery, L.; Leroy, C. *Bull. Soc. Chim. Fr.* **1972**, 4763.
(17) Huckins, J. R.; Rychnovsky, S. D. *J. Org. Chem*. **2003**, *68*, 10135.
(18) Sugiura, M.; Hagio, H.; Hirabayashi, R.; Kobayashi, S. *J. Am. Chem. Soc*., **2001**, *123*, 12510.
(19) Ross, A. G.; Li, X.; Danishefsky, S. J.; *J. Am. Chem. Soc.*, **2012**, *134*, 16080.
(20) Uyanik, M.; Nakashima, D.; Ishihara, K. *Angew. Chem. Int. Ed.* **2012**, *51,* 9093; Gonzalez-Nunez, M. E.; Mello, R.; Olmos, A. G.; Asensio, G.; *J. Org. Chem.* **2006**, *71*, 6432.
(21) Marchand, A. P.; Kumar, V. S.; Hariprakasha, H. K. *J. Org. Chem.* **2001**, *66*, 2072.
(22) Fisher, T. J; Dussault, P. H. *Tetrahedron Letters*, **2010***, 51,* 5615.
